# A New Horizon for Atopic Dermatitis Treatments: JAK Inhibitors

**DOI:** 10.3390/jpm13030384

**Published:** 2023-02-22

**Authors:** Mircea Tampa, Cristina Iulia Mitran, Madalina Irina Mitran, Simona Roxana Georgescu

**Affiliations:** 1 Department of Dermatology, “Carol Davila” University of Medicine and Pharmacy, 020021 Bucharest, Romania; 2 Department of Dermatology, “Victor Babes” Clinical Hospital for Infectious Diseases, 030303 Bucharest, Romania; 3 Department of Microbiology, “Carol Davila” University of Medicine and Pharmacy, 020021 Bucharest, Romania

The article entitled “Application of Janus Kinase Inhibitors in Atopic Dermatitis: An Updated Systematic Review and Meta-Analysis of Clinical Trials” that belongs to the Special Issue, “ Personalized medicine in the field of inflammatory skin diseases”, a collection of articles addressing the current critical issues in the pathogenesis and management of chronic inflammatory skin disorders, represents a valuable work that has contributed to elucidating the role of Janus kinase (JAK) inhibitors as a treatment option for atopic dermatitis (AD), as well as their safety profiles. Tsai et al. analyzed data from 15 randomized clinical trials (7 phase III trials, 7 phase II trials, and 1 phase I trial) that included 4367 patients suffering from AD. They evaluated the efficiency and safety of four oral JAK inhibitors (abrocitinib, baricitinib, gusacitinib, and upadacitinib) and three topical JAK inhibitors (delgocitinib, ruxolitinib, and tofacitinib), and found that JAK inhibitors are superior to placebo in achieving Eczema Area and Severity Index (EASI) 75, as well as good Investigator’s Global Assessment (IGA) and Pruritus Numerical Rating Scale responses in AD patients. The probability of developing adverse events was high, but the reported side effects were tolerable; the most frequently reported side effect was nasopharyngitis. It was observed that the patients who underwent a treatment longer than 12 weeks had a higher risk of developing adverse reactions than those treated with JAK inhibitors for a shorter period [1]. Their study provides very useful data given that they calculated the numbers needed to treat (NNTs) and numbers needed to harm (NNHs) that can guide physicians in clinical practice. Another important strength of their work is that they analyzed 15 multicenter randomized clinical trials in more than 10 countries, offering reliable results that can underlie future studies.

Our knowledge of the various processes by which immune cells communicate to maintain homeostasis and host defense has expanded dramatically as a result of the discovery of the abundance of cytokines encoded in human genomes. Similarly, it was quickly realized that the dysregulated production of cytokines represents a crucial event that underlies the development of numerous dermatological disorders [2]. In chronic inflammatory diseases, proinflammatory cytokines are key pathological drivers. The latest advances in the field highlight that inflammatory cytokines use the JAK/STAT pathway for downstream signal transduction [3]. The JAK/STAT signaling pathway consists of the ligand receptor complexes, JAKs and STATs. The JAK family includes four receptor-associated kinases, JAK1, JAK2, JAK3, and TYK2, and the STAT family consists of seven proteins: STAT1, STAT2, STAT3, STAT4, STAT5a, STAT5b, and STAT6. The JAK/STAT signaling pathway is involved in the normal functioning of cells, including over 50 cytokines and growth factors, and orchestrates processes such as inflammation, tissue regeneration, hematopoiesis, apoptosis, and adipogenesis [4].

JAK inhibitors are small molecules that suppress intracellular signaling mediated by multiple cytokines; thus, numerous studies indicate their effectiveness in many dermatological diseases. Initially, research focused on the study of these molecules in psoriasis [5], but impressive effects have been achieved in other dermatological conditions as well, such as atopic dermatitis, vitiligo, alopecia areata, chilblain lupus, and dermatomyositis [6]. In June 2022, the FDA approved the first systemic treatment for alopecia areata, baricitinib, which is an oral, selective, reversible inhibitor of Janus kinases [7]. A month later, the FDA approved ruxolitinib cream as the first repigmentation therapy for vitiligo [8].

Atopic dermatitis (AD) is a chronic inflammatory condition with a complex pathogenesis based on the interplay between genetic factors, the alteration of the epidermal barrier, abnormalities of the immune response, neuroinflammation, and microbial imbalance [9,10,11,12]. The latest studies have shown that oxidative stress plays an important role in the pathogenesis of AD, a fact that has also been observed in other chronic inflammatory skin conditions [13,14,15,16]. The clinical picture of AD is heterogeneous, and includes numerous signs and symptoms. The cardinal symptom is intense itching, which has important implications for patients’ quality of life [17].

The JAK/STAT pathway modulates multiple immune processes that underlie the pathogenic mechanisms involved in AD, especially Th2 cytokines such as interleukin (IL)-4, IL-5, IL-13, IL-31, and thymic stromal lymphopoietin. It is worth noting that JAK/STAT also modulates the function of the epidermal barrier. JAK/STAT-dependent IL-4 and IL-13 signaling participates in the dysregulation of keratinocyte function in AD, leading to the downregulation of certain components in the skin barrier, such as loricrin, involucrin, and filaggrin. Last, but not least, we should keep in mind that JAK/STAT regulates the activity of peripheral nerves responsible for pruritus, a central symptom in AD [18]. 

As dermatologists, we have faced a plethora of new medication approvals over the last decade. JAK inhibitors have garnered much attention and are being tested as systemic or topical treatment options for AD. A search of the literature in the PubMed database with the keywords “JAK inhibitors” and “atopic dermatitis” retrieved more than 100 articles published in the last year, which demonstrates researchers’ deep concern over the efficacy of these molecules in AD. Numerous trials, both finished and ongoing, have been conducted to assess the use of JAK inhibitors in AD patients. Recently, at the beginning of 2022, the FDA approved abrocitinib and upadacitinib, two oral JAK inhibitors, for the treatment of moderate to severe AD in adults, and other molecules are still being studied [19]. Zhang et al. conducted a systematic review and network meta-analysis (6 randomized clinical trials of JAK inhibitors including 2530 patients) to evaluate the effectiveness of JAK inhibitors in the treatment of AD and highlight which molecules seem to be the most effective. Six JAK inhibitors (abrocitinib, baricitinib, upadacitinib, tofacitinib, ruxolitinib, and delgocitinib) with seventeen different formulations and doses were analyzed. The results of the study confirmed the effectiveness of JAK inhibitors in AD therapy and indicated that upadacitinib 30 mg is the most effective among all included JAK inhibitors; additionally, delgocitinib 3% b.i.d. is more effective compared to other topical JAK inhibitors [20]. In line with this, Lee et al. performed a meta-analysis that evaluated the effectiveness of oral JAK inhibitors by taking into account the dose (abrocitinib (100 and 200 mg), baricitinib (1, 2 and 4 mg), and upadacitinib (15 and 30 mg)) and identified upadacitinib 30 mg as the most effective agent [21].

Several studies have focused on comparing the effectiveness of systemic targeted therapies available for AD. Dupilumab, a human monoclonal antibody that inhibits the signaling of both IL-4 and IL-13, is the first biological agent approved for the treatment of AD patients [22]. Although it has proven effective in many instances, there are also cases in which the response to therapy is not satisfactory, and in this situation alternative therapies are necessary. Recently, in December 2021, the FDA approved tralokinumab, an anti-interleukin-13 monoclonal antibody, to treat moderate to severe AD in adults [23]. The meta-analysis conducted by Sedeh et al. compared the efficacy of dupilumab, tralokinumab, and JAK inhibitors in AD and showed that in monotherapy, upadacitinib 30 mg once daily is the most efficient, using the endpoints EASI-50, EASI-75, and EASI-90. In the case of a combined therapy with topical corticosteroids, dupilumab 300 mg once every other week exhibited the best efficacy based on EASI-50, and patients treated with abrocitinib 200 mg once daily achieved the highest scores for EASI-75 and EASI-90 [24]. Silverberg et al. analyzed the effectiveness of targeted systemic therapies in AD, including abrocitinib, upadacitinib, baricitinib, dupilumab, and tralokinumab without the association of topical corticosteroids, and found that upadacitinib 30 mg daily had the highest efficacy [25].

Interesting results were obtained from the meta-analysis conducted by Li et al., which demonstrated that topical JAK inhibitors are more effective than those administered orally [26]. In this context, a recent systematic review focused on the role of topical JAK inhibitors in AD by analyzing 19 studies that included 3600 participants. The results emphasized the effectiveness of topical delgocitinib, tofacitinib, ruxolitinib, cerdulatinib, and ifidancitinib in patients with AD. The authors also concluded that topical delgocitinib is effective in children [27].

Regarding the long-term adverse events associated with JAK inhibitors, studies have only been conducted and made available for tofacitinib used in the treatment of rheumatoid arthritis [28]. In 2022, the *Journal of the American Academy of Dermatology* published a meta-analysis that highlighted the most common adverse effects associated with the use of JAK inhibitors in patients with AD, namely, gastrointestinal disorders, nasopharyngitis, and headache [29]. However, there are several concerns regarding the safety profiles of JAK inhibitors that can cause laboratory abnormalities and rare severe side effects. In this regard, a recent meta-analysis showed that there is no increased risk of venous thromboembolism in AD patients treated with JAK inhibitors [30]. All these important features should be addressed in patient consultations, patient-specific therapy selection, and patient follow-up.

Currently, the available topical therapies for AD are effective, but adverse effects limit their long-term use in clinical practice. It is well known that the long-term use of topical corticosteroids is associated with striae, skin atrophy, and pigmentation abnormalities. The introduction of a topical phosphodiesterase 4 inhibitor, crisaborole, for AD management has been received with great enthusiasm by the medical community, but its application is limited by a burning sensation [31,32]. Topical calcineurin inhibitors provide a safe and effective alternative to topical corticosteroid use in the treatment of AD; however, patients may be worried about these agents since the FDA implemented a black box warning for them due to the possibility of cancer. Therefore, clinical practitioners continue to follow with interest research into the safety profiles of topical JAK inhibitors. The first topical JAK-1/JAK-2 inhibitor approved by the FDA in September 2021, ruxolitinib, seems to have a similar efficiency to triamcinolone, but does not lead to the adverse effects associated with the use of corticosteroids [32].

In conclusion, JAK inhibitors have revolutionized the treatment of AD patients and also offer fresh perspectives on the pathophysiology of the disease. According to the findings of the current studies, JAK inhibitors have been shown to be effective in AD; however long-term studies to establish their safety profiles are needed. To identify the best alternative with the fewest drawbacks, more extensive research, including head-to-head comparison trials, is needed.
